# AI supported fetal echocardiography with quality assessment

**DOI:** 10.1038/s41598-024-56476-6

**Published:** 2024-03-09

**Authors:** Caroline A. Taksoee-Vester, Kamil Mikolaj, Zahra Bashir, Anders N. Christensen, Olav B. Petersen, Karin Sundberg, Aasa Feragen, Morten B. S. Svendsen, Mads Nielsen, Martin G. Tolsgaard

**Affiliations:** 1 Department of Clinical Medicine, Faculty of Health and Medical Sciences, University of Copenhagen, Copenhagen, Denmark; 2 Center of Fetal Medicine, Department of Obstetrics, Copenhagen University Hospital, Rigshospitalet, Blegdamsvej 9, Dept. 4071, 2100 Copenhagen, Denmark; 3 Copenhagen Academy of Medical Education and Simulation (CAMES), Rigshospitalet, Copenhagen, Denmark; 4 DTU Compute, Technical University of Denmark (DTU), Lyngby, Denmark; 5 Center for Fetal Medicine, Department of Obstetrics, Slagelse Hospital, Slagelse, Denmark; 6 Department of Computer Science, University of Copenhagen, Copenhagen, Denmark

**Keywords:** Medical research, Mathematics and computing, Medical imaging

## Abstract

This study aimed to develop a deep learning model to assess the quality of fetal echocardiography and to perform prospective clinical validation. The model was trained on data from the 18–22-week anomaly scan conducted in seven hospitals from 2008 to 2018. Prospective validation involved 100 patients from two hospitals. A total of 5363 images from 2551 pregnancies were used for training and validation. The model's segmentation accuracy depended on image quality measured by a quality score (QS). It achieved an overall average accuracy of 0.91 (SD 0.09) across the test set, with images having above-average QS scoring 0.97 (SD 0.03). During prospective validation of 192 images, clinicians rated 44.8% (SD 9.8) of images as equal in quality, 18.69% (SD 5.7) favoring auto-captured images and 36.51% (SD 9.0) preferring manually captured ones. Images with above average QS showed better agreement on segmentations (*p* < 0.001) and QS (*p* < 0.001) with fetal medicine experts. Auto-capture saved additional planes beyond protocol requirements, resulting in more comprehensive echocardiographies. Low QS had adverse effect on both model performance and clinician’s agreement with model feedback. The findings highlight the importance of developing and evaluating AI models based on ‘noisy’ real-life data rather than pursuing the highest accuracy possible with retrospective academic-grade data.

## Introduction

Congenital heart disease (CHD) is a prevalent cause of infant mortality and morbidity^1^ occurring in 1.5 per 1000 liveborn children in Denmark^2^. Early detection through prenatal ultrasound, specifically fetal echocardiography, improves perinatal outcomes, and reduces mortality^3,4^.

The detection rate of fetal heart anomalies varies widely and is dependent on multiple factors, including the clinician’s experience level and competence^5–7^, fetal position, and the body mass index (BMI) of the mother, which all affect image quality^8^.

In recent years, computer technology has advanced significantly, and the application of artificial intelligence (AI) presents an opportunity to enhance diagnostic procedures by overcoming some of the inherent limitations of human performance. AI has many benefits, including accuracy, objectivity, and consistency^9^. AI can prevent incomplete examinations and speed up the time-consuming process of taking cardiac measurements, not typically done in routine screening exams^9^. Within many fields of medical imaging, AI is already being used to improve visual diagnosis^10,11^.

AI has shown promise in standard plane detection and segmentation of the fetal heart along with detecting CHDs in fetal medicine using techniques like object detection or segmentation^12–26^. Previous research has emphasized the significance of image quality in ultrasound assessment and developed deep learning models for automatic quality assessment^27–29^. However, there is a notable gap in the literature concerning the lack of multitasking AI systems^9,30^ and the integration of image quality scores into AI segmentation models of the fetal heart^11^. This is particularly noteworthy considering the crucial role image quality plays in CHD detection, directly affecting the detection rates^9^.

Finally, there is need for prospective testing and validation of AI models in this field to ensure future clinical implementation and use^9,31^. Most existing studies have been published on retrospective academic-grade data (subjected to data cleaning and selection), whereas few studies have attempted to identify how AI models perform in real life clinical settings and challenges, evaluated prospectively^32^.

Our aim was to address these gaps by developing an AI model based on screening images for the classification, segmentation, and quality assessment of eight standard planes and key anatomical features in fetal echocardiography and test the model prospectively.

## Results

### Patient and image characteristics

**Figure 1:**
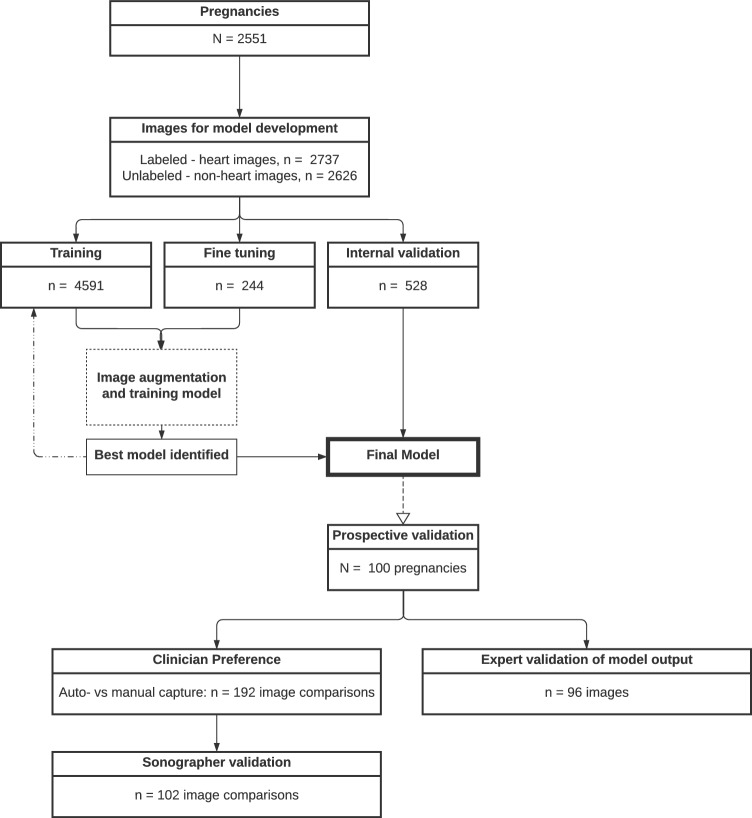
Flowchart summarizing the datasets used in development and validation of the model. *Note*: Unlabeled images—only image level label (which standard plane it is).

For development and internal validation of our AI model 5363 images from a total of 2551 pregnancies were retrieved retrospectively. See Fig. 1 for a flowchart.

A total of 100 full-length ultrasound examinations were recorded for prospective validation. The background characteristics are presented in Table 1. All data was retrieved at gestational age 18–22 weeks.

**Table 1:** Background characteristics for retrospective and prospective data.

	Retrospective data	Prospective data
Patients (N)	2551	100
Images (N)	5363	100 videos (4,688,309 images/video frames)
GA mean (SD)	20.2 weeks (4 days)	20.5 weeks (3.8 days)
Age mean (SD)	31.6 years (4.8)	32.9 years (4.7)
BMI mean (SD)	22.7 (4.1)	24.1 (4.8)

N images under retrospective data refer to both the annotated material and the non-heart planes used to train the model.

4.6 M images under prospective data refer to total number of video frames used for prospective validation.

*BMI* body mass index, *GA* gestational age.

### Model performance

The average accuracy of anatomical structures for each standard plane is illustrated in Table 2. For individual accuracies please refer to Appendix 2. For minimum required anatomical features on each standard plane, we found an overall average accuracy of 0.91 (SD 0.09) for the entire screening test set, and a higher average accuracy of 0.97 (SD 0.03) for the test set with a QS above average, where all relevant anatomical features scored above 0.9 in accuracy. See Appendix 2 for performance scores of the entire tests.

**Table 2:** Accuracy.

Standard plane	No. of annotated images	Average accuracy	Average accuracy
		Mean (SD) entire test set	Mean (SD) test set QS ≥ 6
Situs	300	0.98 (0.04)	1.0 (0.0)
4CV	500	0.95 (0.03)	0.98 (0.03)
3VV	483	0.95 (0.03)	0.97 (0.03)
3VT	308	0.74 (0.13)	0.92 (0.03)
RVOT	441	0.86 (0.07)	0.94 (0.02)
LVOT	222	0.91 (0.02)	0.97 (0.03)
Arch	250	0.97 (0.04)	0.98 (0.02)
VSV	233	0.95 (0.04)	0.99 (0.02)

Mean and SD of minimum required anatomical features’ accuracy pr. plane basis.

*3VT* 3 vessel-trachea view, *3VV* 3 vessel view, *4CV* 4 chamber view, *LVOT* left ventricular outflow tract, *RVOT* right ventricular outflow tract, *QS* quality score, *VSV* ventricular septum view.

### Prospective validation

During the prospective validation, ratings of 192 image comparisons, 24 per standard plane, were carried out by 10–17 raters per plane consisting of a combination of 40.2% fetal medicine experts and 59.8% sonographers. The average preference scores over all eight planes between the auto-capture and manual capture images were 44.8% votes (SD 9.8) for equal quality, 18.69% votes (SD 5.7) for auto-capture images and 36.51% votes (SD 9.0) for manual capture images (X^2^ (2, N = 2571 votes) = 262.84, *p* < 0.001).

The clinical rating panel had a moderate agreement with a multirater kappa value ranging from 0.33 to 0.55 (mean 0.42, SD 0.07). See Appendix 3 for preference results, chi-square results and multirater kappa results per plane basis. In 102 cases the auto-capture or the manual capture image was preferred over the other and for these cases we found an overall QS below average (mean 3.4, SD 2.6). The choice of preference was partly due to a significantly higher QS (favoring auto-capture: U = 953.5, *p* < 0.001, favoring manual capture: U = 14,976, *p* < 0.001) and correct standard plane for the chosen image type, (favoring auto-capture: X^2^ (1, N = 120) = 15.2), *p* < 0.001, favoring manual capture: X^2^ (1, N = 288) = 36.1, *p* < 0.001). Secondly, we found that the preference for auto-capture images consisted of a higher degree of sufficient gain (X^2^ (1, N = 120) = 14.35), *p* < 0.001) and magnification (X^2^ (1, N = 120) = 7.2, *p* = 0.007), whereas for the manual capture images it was only the central positioning of the manual capture image (X^2^ (1, N = 288) = 8.09, *p* = 0.004) which had significant importance.

The expert evaluation of the model’s segmentations and QS assignment on the prospective data showed, in alignment with the model’s performance, a higher agreement with increasing QS (segmentation agreement: X^2^ (1, N = 185) = 34.42, *p* < 0.001, QS agreement: X^2^ (1, N = 185) = 16.55, *p* < 0.001).

Finally, the auto-capture resulted in more complete scans, as the AI saved non-mandatory standard planes; a standard plane of the aortic arch was saved in 31% of the prospective scans with auto-capture, where the sonographer did not save the image.

## Discussion

In this study, we developed an AI model capable of identifying eight standard planes and 28 different anatomical features in fetal echocardiography and assessing image quality. The internal validation of the model demonstrated high accuracy, which increased with image QS above average. However, during the prospective validation, the AI model’s performance was partly inferior to that of experienced clinicians in selecting the best standard planes. Central positioning of relevant anatomical structures was a contributing factor when manual capture was preferred. The auto-captured images were challenged by the fact that these were not meant to be saved by the clinician, and therefore not optimized prior to saving, providing a simple explanation for the lack of central positioning. On the other hand, the preferred auto-capture images had more adequate gain and magnification, which can be explained by the model’s training to save images with the highest QS during the scan. We observed that when auto-capture and manual capture were not deemed equivalent, the general image quality was low. The prospective validation suggests that clinicians exhibited better performance in situations with lower image quality. Even though the model demonstrated high accuracy during internal validation, further requirements were revealed by end-user’s feedback and from the interaction during the prospective clinical validation. While the ultimate goal is to achieve expert-level performance in the demanding setting of live prenatal scans, it may be unrealistic to expect the model to readily attain this level of proficiency, given the differences between the live-scan environment and the training environment. This highlights the importance of prospective clinical validation and of evaluating future AI models based on a broad range of data reflecting real-world variance in quality.

The objective of prenatal ultrasound screening for fetal malformations is to distinguish between normal and abnormal anatomy. The assistance of AI systems during fetal ultrasound examinations have shown to reduce the scan time, and feedback from sonographers indicate that it enables them to focus on the interpretation of relevant images rather than the acquisition and measurement process^33^. Moreover, the use of AI in fetal echocardiography has other benefits, such as making the examination more standardized and performing automatic cardiac measurements^9^. Currently, such measurements are not typically used during screening scans due to the lack of time or expertise. As such, the implementation of automatic cardiac measures based on AI segmentations during screening may improve the detection rate of major CHDs^9^.

While previous research has demonstrated encouraging outcomes in the classification of normal and abnormal hearts^12–14,16^, image quality is a critical factor in the diagnosis of CHDs using both human and AI-based methods. Prior research has emphasized the importance of evaluating image quality to ensure that the AI systems can be relied upon for the downstream target task, such as detecting abnormalities^29^. This is supported by the results in this study, as both the internal and prospective validation showed that the AI segmentation performances were highly dependent on image quality. However, high-quality images may not always be feasible in real-world scenarios during screening scans that involve varying ultrasound equipment and maternal BMI. It is crucial to evaluate and handle image quality issues when creating AI models intended to aid in the diagnostic process. This is emphasized as it is known that CHDs are often missed due to incomplete scans, poor image quality^8^, inadequate standard planes or in some cases the anomaly is visualized but the image is misinterpreted by the clinician^34^ Our study highlights the importance of considering quality when evaluating AI performance, including low-quality real-life data rather than academic-grade high-quality data, and prospective clinical validation to promote transparency in AI systems, build trust, and improve usefulness for clinical end-users.

One limitation to the study is that the AI model is developed and tested solely on a Danish population. However, previous studies have established that AI models based on the same dataset generalize well to other European populations and even to low-resource contexts using small levels of transfer learning^35,36^. Another limitation of the study, often an inevitable bias when working with supervised AI models, is the fact that the segmentations and image QS are based on human manual effort^29^. We alleviated this limitation by building in multiple rounds of discussion and duplicate review during data annotation, and the model segmentation and QS outputs were validated in several prospective settings with different experts.

Additionally, there is a need for a future implementation study of the AI model in which it provides feedback to the clinician on scan completeness and QS, to determine if it improves the overall scan quality and increases the detection rate of fetal heart anomalies by supporting the workflow, quality assessment, and performing automatic measurements. In the context of clinical implementation, fine tuning may be necessary if new clinical guidelines are introduced in clinical practice. Also, advancements in ultrasound technology, especially those significantly altering image appearance (like frequency compounding or coded excitation in challenging patient scenarios), may impact the model's performance.

In conclusion, we have developed a deep learning model trained and validated on screening images from an unselected population that can identify simple and advanced fetal echocardiographic planes. The use of deep learning techniques allows for automated image acquisition and evaluation, which can provide feedback on image quality and the completeness of fetal echocardiography. We discovered that the performance of the model is affected by image quality, and prospective clinical validation is crucial to understand the model's usefulness, strengths, and limitations for future clinical practice. At the same time, our findings question existing practices when developing and evaluating AI models based on academic-grade retrospective data as this may lead to overinflated ideas of model performances and misguide our understanding of when AI models are useful in real-life clinical settings.

## Methods

### Study design and data sources

The study was conducted as a multicenter retrospective study evaluated on prospective data. The study is reported according to the Proposed Requirements for Cardiovascular Imaging-Related Machine Learning Evaluation (PRIME) guidelines^37^. The Danish Health Authorities provided permission for the extraction of ultrasound and outcome data from a large national database of over 600.000 pregnancies for this project. This study has been approved by The Danish Data Protection Agency (Datatilsynet, 12-08-2019, Carl Jacobsens vej 35, 2500 Valby) (protocol no P-2019-310) and The Danish Patient Safety Authority (Styrelsen for Patientsikkerhed, 30-04-2019, Islands Brygge 67, 2300 KBH S) (protocol no 3-3031-2915/1) waiving the need for informed consent.

The collection of prospective data was approved by the Danish Data Protection Agency (protocol no P-2021-570). We developed a deep learning (DL) AI model for fetal echocardiography at 18–22-weeks of pregnancy with the future purpose of assisting the clinician with feedback of image quality, completeness of examination, automatic cardiac measurements, and auto-capture to improve the workflow. The retrospective data consisted of image data from routine fetal echocardiographies performed during the 18–22-week anomaly scan in four Danish regions from 2008 to 2018 comprising a total of more than 25 million ultrasound images from more than 600.000 pregnancies. The training data consisted of 2737 annotated images from 2551 patients. The data was sorted to ensure that images from a single subject were not duplicated and e.g. only included in one of the training, validation and testing groups. For prospective testing 100 planned routine 18–22-week scans were recorded at Rigshospitalet and Slagelse hospital with informed consent from the involved pregnant women in the fall of 2022. All images from the routine 18–22-week scan were eligible for inclusion regardless of the maternal medical history and the potential finding of fetal anomalies for both the prospective and retrospective part of the study. The fetal ultrasound examinations were conducted using General Electrics logiq 7, E6, E8 or E10 machines.

### Ground truth

**Figure 2:**
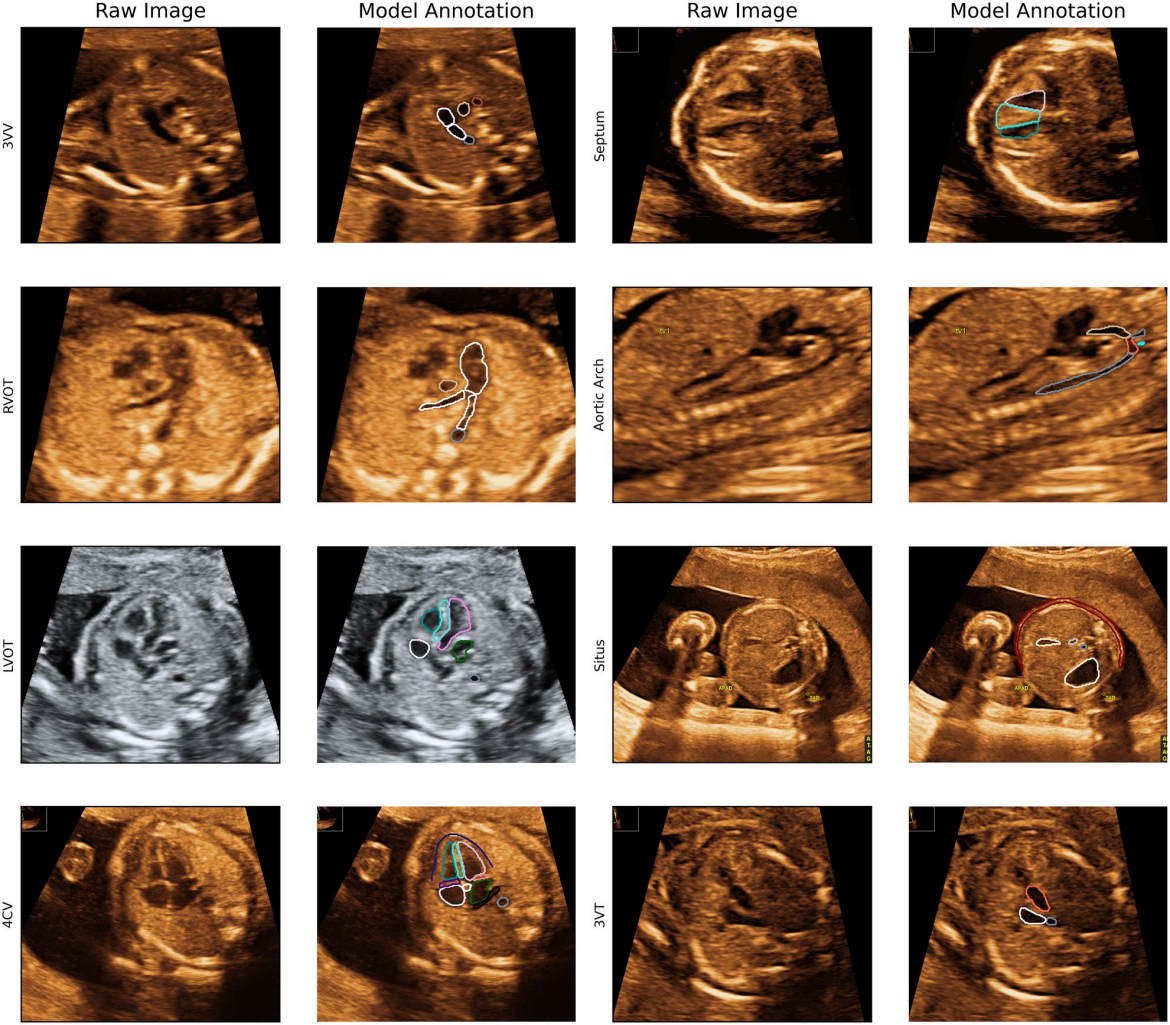
Examples of model annotation per plane. *4CV* 4 chamber view, *3VT* 3 vessel trachea view, *3VV* 3 vessel view, *LVOT* left ventricular outflow tract, *RVOT* ventricular outflow tract, *VSV* ventricular septum view.

Two-dimensional ultrasound images from the retrospective dataset of the 18–22-week pregnancy scan were used to train and validate the model. The annotation included 28 different key anatomical features within eight standard planes of the fetal echocardiography scans, defined by existing international practice guidelines^38^. The standard planes in our model were: the four chamber view (4CV), the ventricular septum view (VSV), the right ventricular outflow tract (RVOT), the left ventricular outflow tract (LVOT), the three-vessel view (3VV), the three-vessel trachea view (3VT), the aortic arch in sagittal plane (AA) and the abdominal situs (Situs). See Fig. 2 for AI annotation and segmentation examples for all planes. The segmentation of relevant anatomical structures serves as a foundation for conducting cardiac biometric measurements and offers transparent explanations to inexperienced clinicians when they have achieved a standard plane or must identify the absent structures required for attainment. Furthermore, an image quality score (QS) was integrated in the model. The QS is a numeric value ranging from 1 to 10 based on the visualization of the structure—10 for perfectly outlined structure and 1 if the outline of the structure was not seen, 5 if the structure was visualized with a degree of shadow or blurriness. Please see Appendix 1 for a thorough description of the QS. The manual annotation was performed in LabelMe©^39^ (version 5.01) by a trainee in obstetrics and Ph.D. fellow in fetal medicine (CAT), in close collaboration with a fetal medicine expert (MGT) at routine quality meetings evaluating any uncertainties.

**Figure 3:**
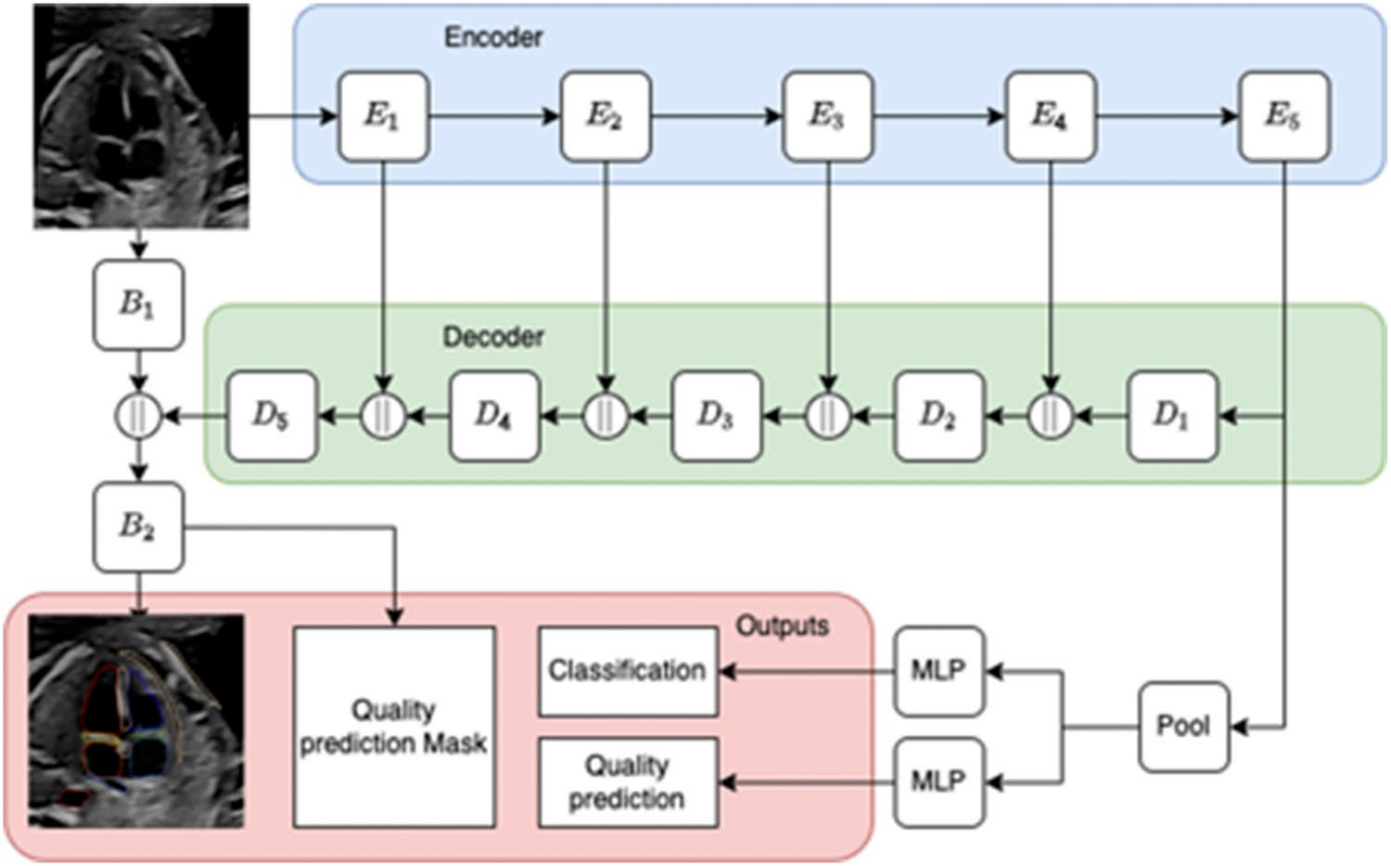
Architecture of the model. Encoder (E), Decoder (D), Block (B), Multi-layer perceptron (MLP). The U-Net architecture used for segmentation. E1–5 are encoders block from RegNetY 1.6Gf architecture, D1–5 are decoder stages, and || represents concatenation. Each decoder stage consists of an upsampling block and two layers with convolution, batch normalisation, and Leaky ReLU activation function. B1 uses the same block architecture but takes grayscale image and outputs 16 channels. B2 is a convolutional layer that outputs 30 channels, 28 of which correspond to segmented classes, and 1 quality score prediction.

Annotations continued until the AI model performed satisfactorily across all image classes. This was reviewed in an iterative fashion by annotating 100 images at a time until the model achieved a 90% accuracy in identifying relevant anatomical features on each standard plane for images with an above-average QS. We chose to have a QS cutoff to evaluate the meaning of the QS both at the internal validation and the prospective validation. See Appendix 1 for a thorough description of the model architecture (depicted in Fig. 3), training and standard plane classification.

Upon testing the model on videos containing many non-heart images, it became apparent that the model trained solely on heart images struggled to distinguish correctly between heart images for segmentation and non-heart images, which should not be segmented. This resulted in false-positive segmentations. To address this limitation, an additional 2626 non-heart images were included in the training dataset. These images had been previously annotated as part of another unpublished study and included only image-level labels that denoted the standard plane on which the image was acquired. The purpose of including these images was to ensure that the segmentation model outputted empty segmentation masks for non-heart images, effectively reducing the false positives.

### Prospective validation

In the second stage of the study the AI model was evaluated in a non-randomized prospective validation study. The aim was to explore how the model works with live ultrasound scans moving from one plane to the other. We collected data and images from 100 18–22-weeks scans.

The AI model automatically collected the standard planes and selected the best images, in terms of appropriate visible anatomy and visualization of the structures—highest image QS. Then images selected by the AI model (auto-capture) were compared to the saved images by the clinician (manual-capture) during the same examination. Standard plane images of 3VV, RVOT and VSV with only a color Doppler image saved were removed since the AI model were not trained on color Doppler images and flow visualization impairs evaluation of the anatomy beneath it. Afterwards an expert panel of sonographers and fetal medicine experts evaluated 24 randomly selected images from each standard plane and each participant noted if they preferred the auto-capture image or manual capture image, or if the quality of the two images were equal. The expert panel was blinded to the origin of the images.

Subsequently two sonographers with more than 5 years of experience went through the images from the rating where the auto-capture image or manual capture image was chosen over the other. They noted if the choice was made due to #1 lack of correct standard plane, defined as missing one or multiple required anatomical features of an standard plane, or #2 poor image optimization in terms of magnification, gain, and centralization of the relevant anatomical features.

To assess the quality of the model’s segmentations and assigned QSs on the prospective data, two fetal medicine consultants evaluated 12 randomly selected images of varying quality from each of the eight standard planes. They evaluated agreement with the segmentations and the assigned QS.

Finally, we evaluated how often the AI saved a useful standard plane from the videos which the sonographer had not saved and documented.

### Statistics

The model output was evaluated by a Dice score regarding prediction of correct anatomy without focusing on the precise outline. This score ranges from 0 to 1 and quantifies the pixel-wise degree of similarity between the model predicted segmentation mask and the ground truth. Whether or not the anatomy was correctly predicted it is treated as a classification task, where True Positive (TP) refers to a Dice score ranging from 0.5 to 1 Dice between prediction and annotation, True Negative (TN) when annotated and segmented areas are both 0. This only happens if there is no expert annotation and model prediction. Furthermore, in this case the False Negative (FN) is defined as Dice below 0.5, and False Positive (FP) as a non-zero predicted area without corresponding expert annotation. The following formula is used to compute the model accuracy: (TP + TN)/(TP + TN + FN + FP).

From the plenum session the frequencies of votes for either AI, clinician, or equal quality were calculated, and a chi-square test was performed to evaluate independence between the three groups. The overall inter-rater agreement was calculated with the Fleiss multirater Kappa.

In the sonographer validation the mean and SD for the QS of all images were calculated, and within each group (favoring auto-capture vs. favoring manual-capture) the mean QS were compared with a Mann–Whitney U test, due to non-normal distribution of data. Each parameter; standard plane, gain, magnification, and central position were scored adequate/not adequate by the sonographers, and chi-squared tests were performed for each parameter within each group to test for independence.

The model was developed and evaluated in Python 3.9.12 using PyTorch 1.10 deep learning library and the statistics for the prospective validation was performed in IBM SPSS statistics 28.0.0.0 (190).

### Supplementary Information

Supplementary Information.

## Data Availability

The data that support the findings of this study are available from the corresponding author upon reasonable request.

## Abbreviations

AA: Aortic arch in sagittal plane
AI: Artificial intelligence
CHD: Congenital heart disease
CNN: Convolutional neural network
DFMF: Danish Fetal Medicine Foundation
DL: Deep learning
FN: False negative
FP: False positive
4CV: Four chamber view
LVOT: Left ventricular outflow tract
QS: Quality score
RVOT: Right ventricular outflow tract
3VV: Three vessel view
3VT: Three vessel trachea view
TN: True negative
TP: True positive
VSV: Ventricular septum view
