# Supplementary material for: AI supported fetal echocardiography with quality assessment
Source: Sci Rep. 2024 Mar 9;14:5809. doi: 10.1038/s41598-024-56476-6 (PMC10925034; doi:10.1038/s41598-024-56476-6)
Supplement: Supplementary file 1 — Supplementary Information. [file 41598_2024_56476_MOESM1_ESM.docx]

**Title: AI Supported Fetal Echocardiography with Quality Assessment**

**Authors:**

Caroline A Taksoee-Vester^1,2,3^

Kamil Mikolaj^4^

Zahra Bashir^1,3,5^

Anders N Christensen^4^

Olav B Petersen^1,2^

Karin Sundberg^2^

Aasa Feragen^4^

Morten BS Svendsen^3^

Mads Nielsen^6^

Martin G Tolsgaard^1,2,3^

1 University of Copenhagen, Dept. of Clinical Medicine, Faculty of Health and Medical Sciences, Denmark

2 Center of Fetal Medicine, Dept. of Gynecology, Fertility and Obstetrics, Copenhagen University Hospital, Rigshospitalet, Denmark

3 Copenhagen Academy of Medical Education and Simulation (CAMES), Rigshospitalet, Denmark

4 DTU Compute, Technical University of Denmark (DTU), Lyngby, Denmark

5 Center for Fetal Medicine, Dept. of Obstetrics, Slagelse Hospital, Denmark

6 University of Copenhagen, Dept. of Computer Science, Denmark

**Corresponding author**

Caroline Amalie Taksoee-Vester

Address: Copenhagen University Hospital, Rigshospitalet, Blegdamsvej 9, Dept. 4071

DK – 2100 Copenhagen

Phone: + 45 20640872

e-mail: [ca_tv@hotmail.com](mailto:ca_tv@hotmail.com)

## Appendix 1

AI Model – Model architecture, Training and Standard Plane Classification.

### Model architecture

In this study, the segmentation task is achieved by combining the encoder with the decoder and adding skip connections between feature layers with matching width and height to form U-Net like architecture ^1^. The block diagram of the proposed model is shown in Figure 2 in the main text. The encoders block E1−5 used in this study is from the RegNetY 1.6Gf architecture ^2^. The decoder stage consists of an up-sampling block and two layers with 3x3 convolution, batch normalization, and dropout, followed by a Leaky ReLU activation function. The dropout probability is set to 0.2, and the number of output channels is set to half of the input. The concatenation along the channel dimension is represented by ||. Furthermore, there are two additional blocks in the proposed architecture, B1 and B2. B1 uses the same block architecture as the decoder stages but takes a grayscale image as input and outputs 16 channels. B2 is a convolutional layer that outputs 30 channels, 28 of which correspond to segmented classes, including background, and 1 quality score prediction.

The last stage of the encoder E5 is passed through a global average pooling layer to obtain an embedding Z. This embedding is processed by two multilayer perceptron (MLP) blocks that output additional class and quality score predictions used in some evaluations. Each MLP block consists of a 3-layer fully connected network with 128 features each and ReLU activation in between.

In this study, two methods for predicting quality scores (QS) were utilized. The first method, denoted as QSemb, used one of the MLP blocks to process the embedding vector Z and output a vector of quality score predictions. The last layer of the MLP did not include any activation function. The quality score of absent structures on a given image was set to 0. As the quality score is partially based on the quality of the boundary of segmented structures, the second method, denoted as QSpx, relied on predicting the QS values for each pixel. To achieve this, the segmentation output was extended with an additional channel treated as a separate regression task. This approach made the quality score prediction meaningful only at the segmentation boundaries. Therefore, the loss originating from pixels away from the boundary was set to 0.

The boundary area and the target value were obtained by performing morphological operations on a given segmentation label. Specifically, each label was dilated with a 5x5 square structuring element to generate M1 and eroded with the same element to generate M2. The boundary mask corresponds to the exclusive OR (XOR) operation between M1 and M2. The same procedure was followed during inference to obtain the boundary masks for each predicted segmentation label. An average of the individual pixels belonging to the mask yielded a quality score for a given anatomical structure. Ultimately the best results were achieved by averaging both quality score prediction methods QS = 0.5 (QSemb + QSpx).

### Training

The retrospective data was split into training (4591), validation (244), and testing (529), ensuring that no patients were included in more than one set. However, it is possible that the same subjects may appear across different standard planes (e.g., 4cv and 3vv may come from the same patient), but this is not considered a problem. Lastly, confounders were not removed because they are rare on heart images compared to the standard planes used for biometry. Before being fed into the model, the images were subject to following transformations: they were converted to grayscale, center-cropped to obtain a square shape with equal width and height, and then resized to 224x224. Moreover, the pixel values were rescaled to a range between 0 and 1. Data augmentation was performed to improve the model’s generalization ability. Specifically, the data was augmented by rotating each image randomly by +/- 30 degrees, applying up to 15 degrees of shear, and performing random cropping. However, random blur was not applied to avoid any potential interactions with the quality score predictions.

The model was trained using the stochastic gradient descent optimizer with a learning rate of 0.01 and weight decay of 1e-6. A batch size of 32 was used, and the model was trained for 3000 epochs. In each epoch, 5000 images were randomly sampled from the dataset. The probability of sampling one of the 8 relevant planes was set to 0.9, while the probability of sampling other planes without a segmentation mask was set to 0.1.

Additionally, multi-task learning was utilized in which the overall loss function incorporated the segmentation ιseg, classification *L_cls_*, and quality score *L_QS_* losses, as indicated in equation 1. It is worth noting that the model’s classification output is not utilized in the final standard plane classification, which is solely based on the segmentation masks. The classification output is incorporated to employ the multi-task learning approach, which has been shown to enhance generalizability [^3^.

$$Loss\text{total} = Loss\text{seg} + Loss\text{cls} + 0.1 (Loss\text{QS\_px}+Loss\text{QS\_emb})$$

The segmentation loss was a combination of Dice (eq. 4) and focal (eq. 3) losses and is expressed in equation 5. The values of γ = 2 and ϵ = 10−5 were set for the parameters. In the following equations, *Y* represents the label, and ŷ denotes the model output.

$$p_{i}=\frac{\exp\left( \hat{Y_{i}} \right)}{\sum_{j}^{N} \exp\left( \hat{Y_{j}} \right)}$$

$$Loss_{focal}=\sum_{n}^{N} Y_{n}\left( 1-p_{n} \right)^{\gamma}\log\left( p_{n} \right)+\left( 1-Y_{n} \right)p_{n}^{\gamma}\log\left( 1-p_{n} \right)$$

$$Loss_{dice}=1-\frac{1}{C}\sum_{c}^{C} \frac{2\sum_{n}^{N} p_{c,n}Y_{c,n}+\epsilon}{\sum_{i}^{N} p_{c,n}+\sum_{n}^{N} Y_{c,n}+\epsilon}$$

$$Loss_{seg}=\frac{1}{2}\left[ Loss_{focal}+Loss_{dice} \right]$$

For the classification task, cross-entropy loss was applied, as demonstrated in equation 6. Both quality prediction losses were defined as mean absolute errors, as shown in equation 7.

$$Loss_{cls}=-\sum_{n}^{N} Y_{i}\log\left( p_{n} \right)+\left( 1-Y_{n} \right)\log\left( 1-p_{n} \right)$$

$$Loss_{qs}=\frac{1}{N}\sum_{n}^{N} \left| Y_{n}-\hat{Y_{n}} \right|$$

### Standard Plane Classification

In this study, we have adopted a standard plane classification approach that prioritizes transparency and interpretability. Specifically, we have based our classification solely on segmentation masks, rather than employing end-to-end classification methods that result in opaque, ”*black box*”, models. Here, the standard planes are defined by the presence of relevant anatomical structures as shown below.

#### Standard plane definitions:

In this study, standard planes in ultrasound images are automatically retrieved from videos using a deep learning model. The model outputs segmentation masks of the anatomical structures and predicts quality scores for each standard plane. The selection of the best standard plane is based on the mean QS of the anatomies present in the given plane. Below is a presentation of a list of standard plane definitions.

Note, for two specific standard planes - the four-chamber view and situs - an additional weighting factor (w) is introduced to prioritize images with more anatomical structures visible. This weighting factor modifies the mean QS calculation for these standard planes. For example, consider a situs standard plane with STB and UVV visible. Its QS will be calculated as:

0.8 * (quality STB + quality UVV) / 2.

For a situs with STB, UVV, Dao, and IVC, the quality score will be calculated as

1. * (quality STB + quality UVV + quality Dao + quality IVC) / 4

By comparing the weighted quality scores, the better standard plane can be selected. The introduction of the weighting factor helps to ensure that images with more relevant anatomical structures are favored, even if their individual QS are slightly lower. This approach enhances the overall quality of the retrieved standard planes.

List of standard planes:

• 4 Chamber View

1. *ω* = 0.7 LV, RV, LA, RA, VS

2. *ω* = 0.95 + Mitral and Tricuspid valves

3. *ω* = 1.0 + Dao, SP and VS angle > 30 deg

• Left Ventricular Outflow Tract - LVOT, VS, RV

• Right Ventricular Outflow Tract - MPA, Aao, RPA, SVC + DA or LPA

• 3 Vessel View - MPA, Aao, SVC

• 3 Vessel Trachea - DA, AA, Dao

• Septum View - RV, LV, VS and VS angle < 30 deg

• Aortic Arch in sagittal plane - Aao, AA, Dao

• Situs

1. *ω* = 0.8 STB, Uvv

2. *ω* = 1.0 STB, Uvv, Dao, IVC

| Abbreviations |  |
| --- | --- |
| Ascending aorta – Aao | Multilayer perceptron – MLP |
| Aortic arch – AA | Right atrium – RA |
| Descending aorta – Dao | Right pulmonary artery – RPA |
| Ductus arteriosus – DA | Right ventricle - RV |
| Inferior vena cava – IVC | Septum primum – SP |
| Left atrium – LA | Stomach bubble – STB |
| Left pulmonary artery – LPA | Superior vena cava – SVC |
| Left ventricle - LV | Umbilical vein – UVV |
| Left ventricular outflow tract – LVOT | Ventricular septum – VS |
| Main pulmonary artery – MPA | Quality score – QS |

### References

1 Ronneberger O, Fischer P, Brox T. U-net: Convolutional networks for biomedical image segmentation. In: Lecture Notes in Computer Science (including subseries Lecture Notes in Artificial Intelligence and Lecture Notes in Bioinformatics). 2015. DOI:10.1007/978-3-319-24574-4_28.

2 Radosavovic I, Kosaraju RP, Girshick R, He K, Dollár P. Designing Network Design Spaces. 2020; published online March 30. http://arxiv.org/abs/2003.13678.

3 Caruana R. Multitask learning. *Mach Learn* 1997; **28**: 41–75.

### Disclosure

This manuscript uses methods described in another publication's supplementary material (the model development). The other publication is centered around a particular use case of the model and pursues objectives distinct from prospective and clinical validation

## Appendix 2 Model Performance scores per plane basis

1. Table A Results from the entire test set
2. Table B Results from the test set with a QS => 6

**Table A**

| Anatomical structure | True Positive | True Negative | False Positive | False Negative | Accuracy | Sensitivity | Specificity |
| --- | --- | --- | --- | --- | --- | --- | --- |
| 4CV |  |  |  |  |  |  |  |
| Right Ventricle | 109 | 0 | 0 | 4 | 0.96 | 0.96 | 0 |
| Left Ventricle | 108 | 0 | 0 | 5 | 0.96 | 0.96 | 0 |
| Right Atrium | 108 | 0 | 0 | 5 | 0.96 | 0.96 | 0 |
| Left Atrium | 110 | 0 | 0 | 3 | 0.97 | 0.97 | 0 |
| Ventricular Septum | 110 | 0 | 0 | 3 | 0.97 | 0.97 | 0 |
| Tricuspid Valve | 102 | 2 | 2 | 7 | 0.92 | 0.94 | 0.5 |
| Mitral Valve | 98 | 2 | 2 | 11 | 0.88 | 0.90 | 0.5 |
| Descending Aorta | 105 | 2 | 2 | 4 | 0.95 | 0.96 | 0.5 |
|  |  |  |  |  |  |  |  |
| VSV |  |  |  |  |  |  |  |
| Right Ventricle | 71 | 0 | 0 | 6 | 0.92 | 0.92 | 0 |
| Left Ventricle | 73 | 0 | 0 | 4 | 0.95 | 0.95 | 0 |
| Ventricular Septum | 76 | 0 | 0 | 1 | 0.99 | 0.99 | 0 |
|  |  |  |  |  |  |  |  |
| LVOT |  |  |  |  |  |  |  |
| Right Ventricle | 80 | 0 | 4 | 5 | 0.90 | 0.94 | 0 |
| LVOT | 80 | 0 | 0 | 9 | 0.90 | 0.90 | 0 |
| Descending Aorta | 68 | 4 | 5 | 12 | 0.81 | 0.85 | 0.4 |
| Left Atrium | 73 | 3 | 0 | 13 | 0.85 | 0.85 | 1 |
| Ventricular Septum | 83 | 0 | 0 | 6 | 0.93 | 0.93 | 0 |
|  |  |  |  |  |  |  |  |
| RVOT |  |  |  |  |  |  |  |
| Main Pulmonary Artery | 87 | 0 | 0 | 4 | 0.96 | 0.96 | 0 |
| Ductus Arteriosus | 35 | 40 | 7 | 9 | 0.82 | 0.79 | 0.85 |
| Right Pulmonary Artery | 77 | 0 | 0 | 14 | 0.85 | 0.85 | 0 |
| Left Pulmonary Artery | 32 | 40 | 4 | 15 | 0.79 | 0.68 | 0.91 |
|  |  |  |  |  |  |  |  |
| 3VV |  |  |  |  |  |  |  |
| Main Pulmonary Artery | 106 | 0 | 0 | 5 | 0.96 | 0.96 | 0 |
| Ascending Aorta | 108 | 0 | 0 | 3 | 0.97 | 0.97 | 0 |
| Superior Vena Cava | 102 | 0 | 2 | 7 | 0.92 | 0.94 | 0 |
|  |  |  |  |  |  |  |  |
| 3VT |  |  |  |  |  |  |  |
| Ductus arteriosus | 43 | 0 | 0 | 22 | 0.66 | 0.66 | 0 |
| Arcus aorta | 40 | 0 | 0 | 25 | 0.62 | 0.62 | 0 |
| Descending aorta | 49 | 2 | 0 | 14 | 0.78 | 0.78 | 1 |
| superior vena cava | 59 | 0 | 0 | 6 | 0.91 | 0.91 | 0 |
|  |  |  |  |  |  |  |  |
| AA |  |  |  |  |  |  |  |
| ascending aorta | 52 | 0 | 0 | 4 | 0.93 | 0.93 | 0 |
| arcus aorta | 55 | 0 | 0 | 1 | 0.98 | 0.98 | 0 |
| descending aorta | 56 | 0 | 0 | 0 | 1.0 | 1.0 | 0 |
|  |  |  |  |  |  |  |  |
| Situs |  |  |  |  |  |  |  |
| Stomach bubble | 57 | 0 | 0 | 0 | 1.0 | 1.0 | 0 |
| umbilical vein | 57 | 0 | 0 | 0 | 1.0 | 1.0 | 0 |
| Descending aorta | 57 | 0 | 0 | 0 | 1.0 | 1.0 | 0 |
| inferior vena cava | 53 | 0 | 0 | 0 | 0.93 | 0.93 | 0 |

4 Chamber view (4CV), Ventricular septum view (VSV), Left ventricular outflow tract (LVOT), ventricular outflow tract (RVOT), 3 vessel view (3VV), 3 vessel trachea view (3VT), Aortic arch (AA).

##

**Table B**

| Anatomical structure | True Positive | True Negative | False Positive | False Negative | Accuracy | Sensitivity | Specificity |
| --- | --- | --- | --- | --- | --- | --- | --- |
| 4CV |  |  |  |  |  |  |  |
| Right Ventricle | 46 | 0 | 0 | 1 | 0.98 | 0.98 | 0 |
| Left Ventricle | 46 | 0 | 0 | 1 | 0.98 | 0.98 | 0 |
| Right Atrium | 46 | 0 | 0 | 1 | 0.98 | 0.98 | 0 |
| Left Atrium | 47 | 0 | 0 | 0 | 1.0 | 1.0 | 0 |
| Ventricular Septum | 47 | 0 | 0 | 0 | 1.0 | 1.0 | 0 |
| Tricuspid Valve | 43 | 1 | 0 | 3 | 0.93 | 0.93 | 1.0 |
| Mitral Valve | 44 | 1 | 0 | 2 | 0.95 | 0.96 | 1.0 |
| Descending Aorta | 46 | 0 | 1 | 0 | 0.98 | 1.0 | 0 |
|  |  |  |  |  |  |  |  |
| VSV |  |  |  |  |  |  |  |
| Right Ventricle | 32 | 0 | 0 | 1 | 0.97 | 0.97 | 0 |
| Left Ventricle | 33 | 0 | 0 | 0 | 1.0 | 1.0 | 0 |
| Ventricular Septum | 33 | 0 | 0 | 0 | 1.0 | 1.0 | 0 |
|  |  |  |  |  |  |  |  |
| LVOT |  |  |  |  |  |  |  |
| Right Ventricle | 37 | 0 | 1 | 0 | 0.97 | 1.0 | 0 |
| LVOT | 36 | 0 | 0 | 2 | 0.95 | 0.95 | 0 |
| Descending Aorta | 36 | 1 | 1 | 0 | 0.97 | 1.0 | 0.5 |
| Left Atrium | 37 | 1 | 0 | 0 | 1.0 | 1.0 | 1 |
| Ventricular Septum | 38 | 0 | 0 | 0 | 1.0 | 1.0 | 0 |
|  |  |  |  |  |  |  |  |
| RVOT |  |  |  |  |  |  |  |
| Main Pulmonary Artery | 37 | 0 | 0 | 2 | 0.95 | 0.95 | 0 |
| Ductus Arteriosus | 17 | 19 | 1 | 2 | 0.92 | 0.89 | 0.95 |
| Right Pulmonary Artery | 36 | 0 | 0 | 3 | 0.92 | 0.92 | 0 |
| Left Pulmonary Artery | 18 | 19 | 0 | 2 | 0.95 | 0.9 | 1.0 |
|  |  |  |  |  |  |  |  |
| 3VV |  |  |  |  |  |  |  |
| Main Pulmonary Artery | 30 | 0 | 0 | 1 | 0.97 | 0.97 | 0 |
| Ascending Aorta | 31 | 0 | 0 | 0 | 1.0 | 1.0 | 0 |
| Superior Vena Cava | 29 | 0 | 0 | 2 | 0.94 | 0.94 | 0 |
|  |  |  |  |  |  |  |  |
| 3VT |  |  |  |  |  |  |  |
| Ductus arteriosus | 22 | 0 | 0 | 2 | 0.92 | 0.92 | 0 |
| Arcus aorta | 22 | 0 | 0 | 2 | 0.92 | 0.92 | 0 |
| Descending aorta | 20 | 1 | 0 | 3 | 0.88 | 0.87 | 1 |
| superior vena cava | 23 | 0 | 0 | 1 | 0.96 | 0.96 | 0 |
|  |  |  |  |  |  |  |  |
| AA |  |  |  |  |  |  |  |
| ascending aorta | 30 | 0 | 0 | 1 | 0.97 | 0.97 | 0 |
| arcus aorta | 30 | 0 | 0 | 1 | 0.97 | 0.97 | 0 |
| descending aorta | 31 | 0 | 0 | 0 | 1.0 | 1.0 | 0 |
|  |  |  |  |  |  |  |  |
| Situs |  |  |  |  |  |  |  |
| Stomach bubble | 23 | 0 | 0 | 0 | 1.0 | 1.0 | 0 |
| umbilical vein | 23 | 0 | 0 | 0 | 1.0 | 1.0 | 0 |
| Descending aorta | 23 | 0 | 0 | 0 | 1.0 | 1.0 | 0 |
| inferior vena cava | 23 | 0 | 0 | 0 | 1.0 | 1.0 | 0 |

Note: When True Negatives (TN) equals zero, the specificity, calculated as Specificity = TN / (TN + False Positives, FP), will be zero as well. This occurs because, due to our partitioning of results on a per-plane basis, certain anatomical structures such as the atriums and ventricles in the 4CV view will consistently be present. Consequently, the specificity for these "required" anatomies on a given scanning plane will consistently be zero.

## Appendix 3.

## Prospective validation results.

## Multi-rater kappa values per plane basis

## Preference votes and Chi square test results per plane basis.

1. Multirater Kappa Values.

| Standard plane | Kappa | Standard Error | Z | Sig. | 95 % confidence interval |
| --- | --- | --- | --- | --- | --- |
| Situs | 0.329 | 0.017 | 19.672 | 0.000 | 0.297-0.362 |
| 4CV | 0.552 | 0.013 | 41.208 | 0.000 | 0.526-0.579 |
| 3VV | 0.399 | 0.014 | 28.145 | 0.000 | 0.372-0.427 |
| 3VT | 0.414 | 0.017 | 23.902 | 0.000 | 0.380-0.448 |
| RVOT | 0.342 | 0.017 | 19.749 | 0.000 | 0.308-0.376 |
| LVOT | 0.442 | 0.023 | 19.289 | 0.000 | 0.397-0.486 |
| Arch | 0.490 | 0.018 | 26.921 | 0.000 | 0.454-0.526 |
| Septum | 0.423 | 0.019 | 22.365 | 0.000 | 0.386-0.460 |

1. Preference votes and Chi-square test results

| Standard plane | Preference | Frequency | Observed N | Expected N | Residual | Chi-square | df | Asymp. Sig. |
| --- | --- | --- | --- | --- | --- | --- | --- | --- |
| Situs | AI | 21.15% | 66 | 104 | -38 |  |  |  |
|  | Expert | 41.35% | 129 | 104 | 25 |  |  |  |
|  | Equal | 37.5% | 117 | 104 | 13 |  |  |  |
|  | Total | 100% | 312 |  |  | *21.519* | *2* | *<.001* |
| 4CV | AI | 21.3% | 86 | 134.7 | -48.7 |  |  |  |
|  | Expert | 46.3% | 187 | 134.7 | 52.3 |  |  |  |
|  | Equal | 32.4% | 131 | 134.7 | -3.7 |  |  |  |
|  | Total | 100% | 404 |  |  | *38.025* | *2* | *<.001* |
| 3VV | AI | 26.4% | 95 | 120 | -25 |  |  |  |
|  | Expert | 36.1% | 130 | 120 | 10 |  |  |  |
|  | Equal | 37.5% | 135 | 120 | 15 |  |  |  |
|  | Total | 100% | 360 |  |  | *7.917* | *2* | *0.019* |
| 3VT | AI | 15.7% | 49 | 104 | -55 |  |  |  |
|  | Expert | 32.05% | 100 | 104 | -4 |  |  |  |
|  | Equal | 52.25% | 163 | 104 | 59 |  |  |  |
|  | Total | 100% | 312 |  |  | *62.712* | *2* | *<.001* |
| RVOT | AI | 13.54% | 44 | 108.3 | -64.3 |  |  |  |
|  | Expert | 33.23% | 108 | 108.3 | -.3 |  |  |  |
|  | Equal | 53.23% | 173 | 108.3 | 64.7 |  |  |  |
|  | Total | 100% | 325 |  |  | *76.806* | *2* | *<.001* |
| LVOT | AI | 15% | 36 | 80 | -44 |  |  |  |
|  | Expert | 49.17% | 118 | 80 | 38 |  |  |  |
|  | Equal | 35.83% | 86 | 80 | 6 |  |  |  |
|  | Total | 100% | 240 |  |  | *42.700* | *2* | *<.001* |
| Arch | AI | 10.9% | 34 | 104 | -70 |  |  |  |
|  | Expert | 33% | 103 | 104 | -1 |  |  |  |
|  | Equal | 56.1% | 175 | 104 | 71 |  |  |  |
|  | Total | 100% | 312 |  |  | *95.596* | *2* | *<.001* |
| Septum | AI | 25.5% | 78 | 102 | -24 |  |  |  |
|  | Expert | 20.9% | 64 | 102 | -38 |  |  |  |
|  | Equal | 53.6% | 164 | 102 | 62 |  |  |  |
|  | Total | 100% | 306 |  |  | *57.490* | *2* | *<.001* |
